# Reconstruction of bovine spermatozoa substances distribution and morphological differences between Holstein and Korean native cattle using three-dimensional refractive index tomography

**DOI:** 10.1038/s41598-019-45174-3

**Published:** 2019-06-19

**Authors:** Hao Jiang, Jeong-woo Kwon, Sumin Lee, Yu-Jin Jo, Suk Namgoong, Xue-rui Yao, Bao Yuan, Jia-bao Zhang, Yong-Keun Park, Nam-Hyung Kim

**Affiliations:** 1 Department of Animal Sciences, Chungbuk National University, Cheong-Ju, Chungbuk Republic of Korea; 2 Tomocube, Inc., 48, Yuseong-daero 1184beon-gil, Yuseong-Gu, Daejeon 34051 Republic of Korea; 3 Department of Physics, Korea Advanced Institute of Science and Technology (KAIST), Daejeon, 34141 Republic of Korea; 4 KAIST Institute for Health Science and Technology, KAIST, Daejeon, 34141 Republic of Korea; 5 College of Animal Science, Jilin University, Changchun, China

**Keywords:** Image processing, Cellular imaging, Imaging and sensing

## Abstract

Measurements of the three-dimensional (3D) structure of spermatozoon are crucial for the study of developmental biology and for the evaluation of *in vitro* fertilization. Here, we present 3D label-free imaging of individual spermatozoon and perform quantitative analysis of bovine, porcine, and mouse spermatozoa morphologies using refractive index tomography. Various morphological and biophysical properties were determined, including the internal structure, volume, surface area, concentration, and dry matter mass of individual spermatozoon. Furthermore, Holstein cows and Korean native cattle spermatozoa were systematically analyzed and revealed significant differences in spermatozoa head length, head width, midpiece length, and tail length between the two breeds. This label-free imaging approach provides a new technique for understanding the physiology of spermatozoa.

## Introduction

The structure of mammalian spermatozoa (sperm) is strongly related to their functions, which include sperm capacitation, acrosome reaction, and the fertilization process. Various imaging approaches have been utilized to examine spermatozoa. For example, their adaptability to high-viscosity environments^1,2^, acrosome reaction, structural changes during maturation and capacitation^3–5^, movement patterns^6,7^, X/Y chromosome bearing ability^8^, motility, apoptosis^9^, and fertilization ability^10^ have all been discovered by analyzing spermatozoa morphology^11^. Detailed analyses of spermatozoa head, flagella, acrosome, perinuclear theca, mitochondrion, DNA status, and plasma have also been undertaken using various two-dimensional (2D) imaging techniques^12,13^.

Despite its importance, label-free three-dimensional (3D) imaging of live spermatozoa has not been performed, mainly due to the limitations of the imaging technique. Electron microscopic techniques can provide high-resolution imaging; however, they cannot be used on live spermatozoa. Conventional optical imaging approaches, including confocal fluorescence microscopy, have been used previously to study spermatozoa. However, these approaches require the use of exogenous labeling agents, such as fluorescent proteins or dyes, which interfere with the intrinsic pigments^14^ and alter the physiological states of spermatozoa cells^15^, and may cause photo-bleaching or photo-toxicity^16^. The use of such labeling agents is strongly prohibited during *in vitro* fertilization. Therefore, quantitative morphological information such as cytoplasmic density distribution is difficult to retrieve using conventional approaches.

Recently, quantitative phase imaging (QPI) and tomographic phase microscopy (TPM) have emerged as label-free imaging techniques for the study of live cells, with promising directions envisaged for biomedical research^17–20^. Exploiting the principle of laser interferometry, the 2D QPI technique measures the optical phase delay of unlabeled cells. Optical phase delay is defined as the integration of the refractive index (RI) along an optical path; the topographic information (optical thickness) of the sample is retrieved from 2D QPI under the assumption and prior information that the RI distribution inside a sample is homogenous. Because the RI is an intrinsic optical imaging contrast technique, TPM and QPI enable quantitative and label-free imaging of diatoms, bacteria, cells, tissues, and semitransparent objects^21–24^. Recently, 2D QPI techniques have been used to map the optical thickness of individual spermatozoa cells^25–27^. Although these studies have provided the potential for label-free assessments of spermatozoa, the technique is limited to 2D imaging and does not provide 3D information regarding internal structures. Despite its important applications, label-free 3D RI tomography of individual spermatozoon has not been fully investigated.

In the present study, we measured 3D RI tomograms of individual bovine spermatozoa using the 3D QPI technique of optical diffraction tomography (ODT) or holotomography (HT). As an optical analogy to X-ray computed tomography (CT), ODT can construct and analyze multiple 2D optical phase delay maps of a sample containing various illumination angles, from which a 3D RI tomogram can be reconstructed^28^. From the measured RI tomograms, the characteristic morphology of the spermatozoa was quantitatively retrieved and systematically analyzed. To demonstrate the applicability of the present method, Holstein (HO) and Korean native cattle (KN) spermatozoa were measured and their parameters analyzed. Statistical significance tests were performed to measure differences in the spermatozoa head length, head width, midpiece length, and tail length between HO cows and KN cattle. We also investigated the morphological and chemical properties of spermatozoa substances.

## Results

### General 2D and 3D imaging of bovine, porcine, and mouse spermatozoa

**Figure 1:**
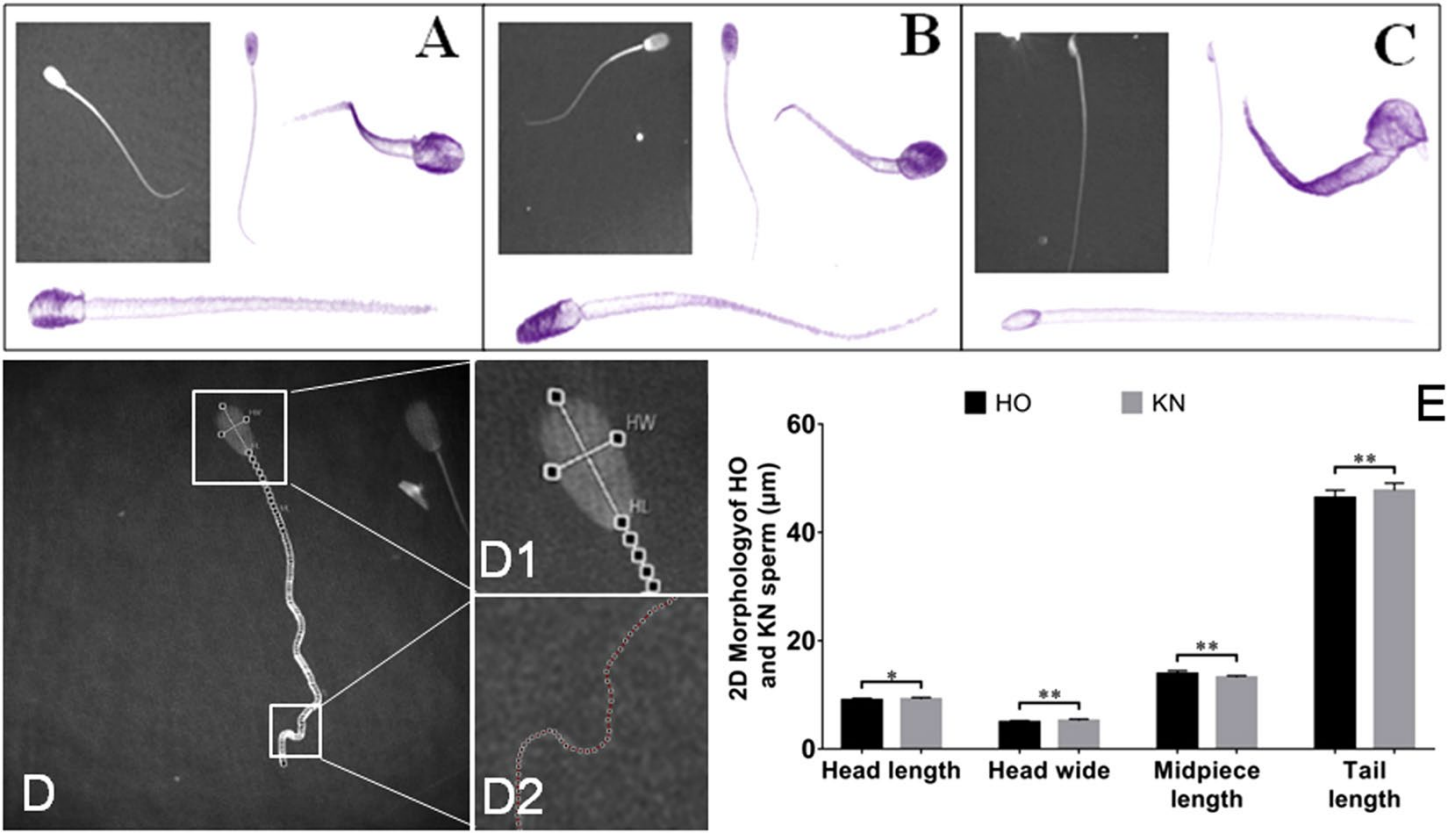
3D RI tomograms of bovine, porcine and mouse sperms and 2D morphology of HO and KN bovine sperm. (**A–C**) 3D RI tomograms of bovine, porcine and mouse sperms. Representative sperm cell image of (**A**) a HO bull; (**B**) a Large White pig; (**C**) an ICR mouse. Cell membranes of the sperm cells were visualized by applying pseudo colouring to the region with RI values of 1.3400 to 1.3850. (**D**) 2D optical phase images of sperm. D1 and D2 are the enlarged images of the selected areas of spermhead and tail, respectively. (**E**) Differences in 2D morphological parameters of the sperms between the HO and KN cattle.

To non-invasively investigate the 3D morphology of spermatozoa cells, the RI tomograms of individual spermatozoa cells from bovine, porcine, and mice were measured using a commercial HT instrument, which could continuously monitor the spermatozoa (Video 1–6). The representative cell RI tomograms and 2D optical phase images are shown in Fig. 1. The 2D optical phase images and 3D RI tomograms of the spermatozoa cells clearly visualized their distinct anatomical structures. The 2D optical phase images showed the characteristic morphology of the mouse spermatozoa, which was different from that of the bovine and porcine spermatozoa. However, the morphologies of the bovine and porcine spermatozoa were not easily distinguishable using the 2D optical phase images. In contrast, the 3D RI tomograms of the spermatozoa showed a significant difference in the morphology of individual cells, distinguishing all three groups. The sperm head of the bovine (Fig. 1A), porcine (Fig. 1B), and mouse (Fig. 1C) showed noticeably different sphericity. The head of the bovine spermatozoa had the highest sphericity, followed by the porcine spermatozoa, and finally the mouse spermatozoa. The 3D tomograms of spermatozoa from the three groups presented bilateral symmetry when seen from the outside. The midpiece portion of the porcine and mouse spermatozoa were more pronounced than those of the bovine spermatozoa. The head shape of the bovine and mouse spermatozoa had distinct back, abdominal, left, and right sides, and the front part of the mouse spermatozoan head was eagle beak-shaped.

### Morphological analysis of different bovine spermatozoa

To further investigate the morphology of individual spermatozoon, we assessed the spermatozoa of HO cows and KN cattle and conducted an in-depth morphological analysis (Fig. 1D,E). The 2D optical phase images enabled the length and width of the head, and length of the midpiece and tail of the HO cow and KN cattle spermatozoa to be analyzed. The 2D morphological analysis results showed that the head lengths of the HO cow and KN cattle spermatozoa were 9.053 ± 0.310 µm and 9.229 ± 0.297 µm, respectively (P < 0.05); the head widths were 4.954 ± 0.268 µm and 5.241 ± 0.297 µm, respectively (P < 0.01); the midpiece lengths were 13.926 ± 0.547 µm and 13.245 ± 0.299 µm, respectively (P < 0.01); and the tail lengths were 46.415 ± 1.386 µm and 47.750 ± 1.373 µm (P < 0.01), respectively.

**Figure 2:**
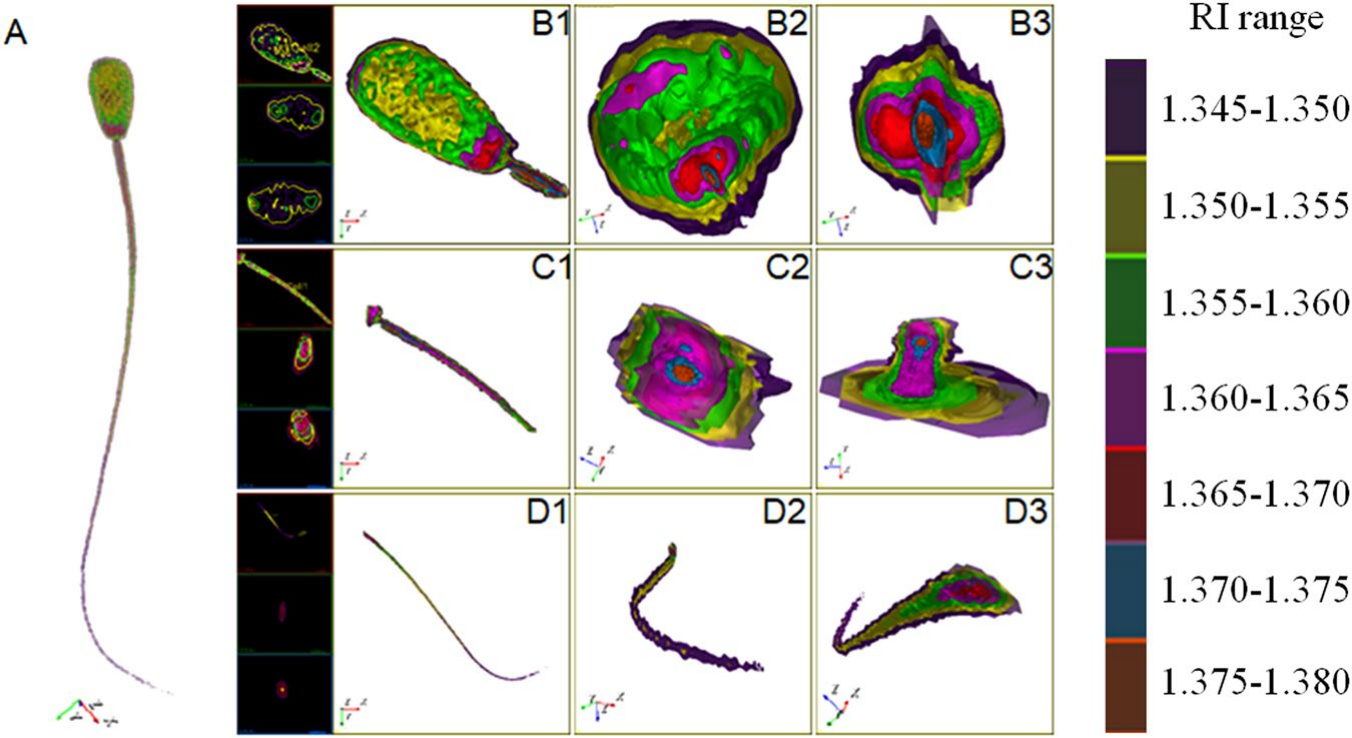
3D model construction of bovine sperm based on gradient RI. (**A**) 3D RI tomogram of whole bovine sperm, visualised at the different RI ranges. B1 to B3: the sperm head viewed at various perspective angles. C1 to C3: the sperm midpiece viewed at various perspective angles. D1 to D3: the sperm tail viewed at various perspective angles. Cross-sectional images of B1, C1, and D1, along with the x-y, the x-z, and the z planes, from top to bottom. Dry matter mass with different RI ranges were digitally colour-coded.

To perform volumetric analysis of the HO cow and KN cattle spermatozoa, we first obtained ten independent measurements from one bovine spermatozoon and visualized the reconstructed RI tomograms of the entire spermatozoa using the detectable RI range, which showed high specificity and accuracy of substance distribution between 1.345 and 1.380, and a gradient of 0.005 (Fig. 2 and Supplementary Table S1). Then, by optimizing the parameter settings based on the entire RI range (1.345 to 1.380), we found that three RI ranges, namely, RI-I, RI-II, and RI-III, effectively represented the overall cell morphology and internal structures, including the approximate location of the mitochondria. We used these parameter settings for subsequent analyses.

### Biophysical analysis of the bovine spermatozoa

To perform biophysical analysis of the individual cattle spermatozoon, we retrieved the biophysical parameters from the measured RI tomograms, including volume, surface area, concentration, and dry mass. Because the RI value of the cell cytoplasm is linearly proportional to its concentration^29^, the concentration of spermatozoa can be obtained from the measured RI tomogram. In addition, the spermatozoa dry mass can be calculated from the retrieved concentration and the measured cell volume.

As shown in Supplementary Fig. S1, the overall volumes of HO cow and KN cattle spermatozoa with maximum detectable RI_1.335–1.385_ were 145.3 ± 2.04 µm^3^ and 156.3 ± 1.71 µm^3^, respectively (P < 0.01); surface areas were 352.5 ± 3.62 µm^2^ and 369.9 ± 3.48 µm^2^, respectively (P < 0.01); dry mass concentrations were 72.5 ± 0.05 fg/µm^3^ and 68.1 ± 0.04 fg/µm^3^, respectively (P < 0.01); and dry matter masses were 10.49 ± 0.14 pg and 10.74 ± 0.12 pg, respectively.

To further analyze the components of the spermatozoa, the individual spermatozoon were divided into three components: head, midpiece, and tail, and quantitative imaging analysis was performed on each component (Table 1 and Supplementary Fig. S2). No significant differences were found among the dry mass of the sperm head, midpiece, and tail. For the substances of sperm head, HO cattle had a much higher concentration and sphericity but a smaller volume and surface area than that of KN cattle. For the substances of sperm midpiece, HO cattle had a larger volume and higher concentration than that of KN cattle, whereas no significant difference was found in surface area. For the substances of sperm tail, no significant differences were observed in volume, surface area, and concentration between the two breeds.

**Table 1:** The 3D Morphology differences of subparts between HO and KN sperm with RI_1.340–1.385_.

Subpart	Specie	Volume (μm^3^)	Surface area (μm^2^)	Concentration (fg/μm^3^)	Dry mass (pg)	Sphericity
Head	HO	94.18 ± 1.28^a^	143.3 ± 2.08^A^	75.9 ± 0.89^A^	6.496 ± 0.11	0.67 ± 0.01^A^
	KN	99.00 ± 1.50^b^	174.7 ± 3.95^B^	72.3 ± 0.92^B^	6.5538 ± 0.10	0.56 ± 0.02^B^
Midpiece	HO	20.61 ± 0.43^A^	77.67 ± 0.75	136.5 ± 6.76^A^	1.94 ± 0.05	—
	KN	18.40 ± 0.44^B^	77.20 ± 1.04	111.9 ± 4.00^B^	1.84 ± 0.05	
Tail	HO	8.94 ± 0.65	78.20 ± 1.08	153.5 ± 15.18	1.282 ± 0.03	—
	KN	9.50 ± 0.63	77.43 ± 1.30	166.4 ± 14.47	1.228 ± 0.02	

Significant differences are represented with different lower-case letters (P < 0.05) and different capital letters (P < 0.01) between HO and KN. — Represents undetectable.

### 3D tomography analysis of HO cow and KN cattle spermatozoa inner structure

**Figure 3:**
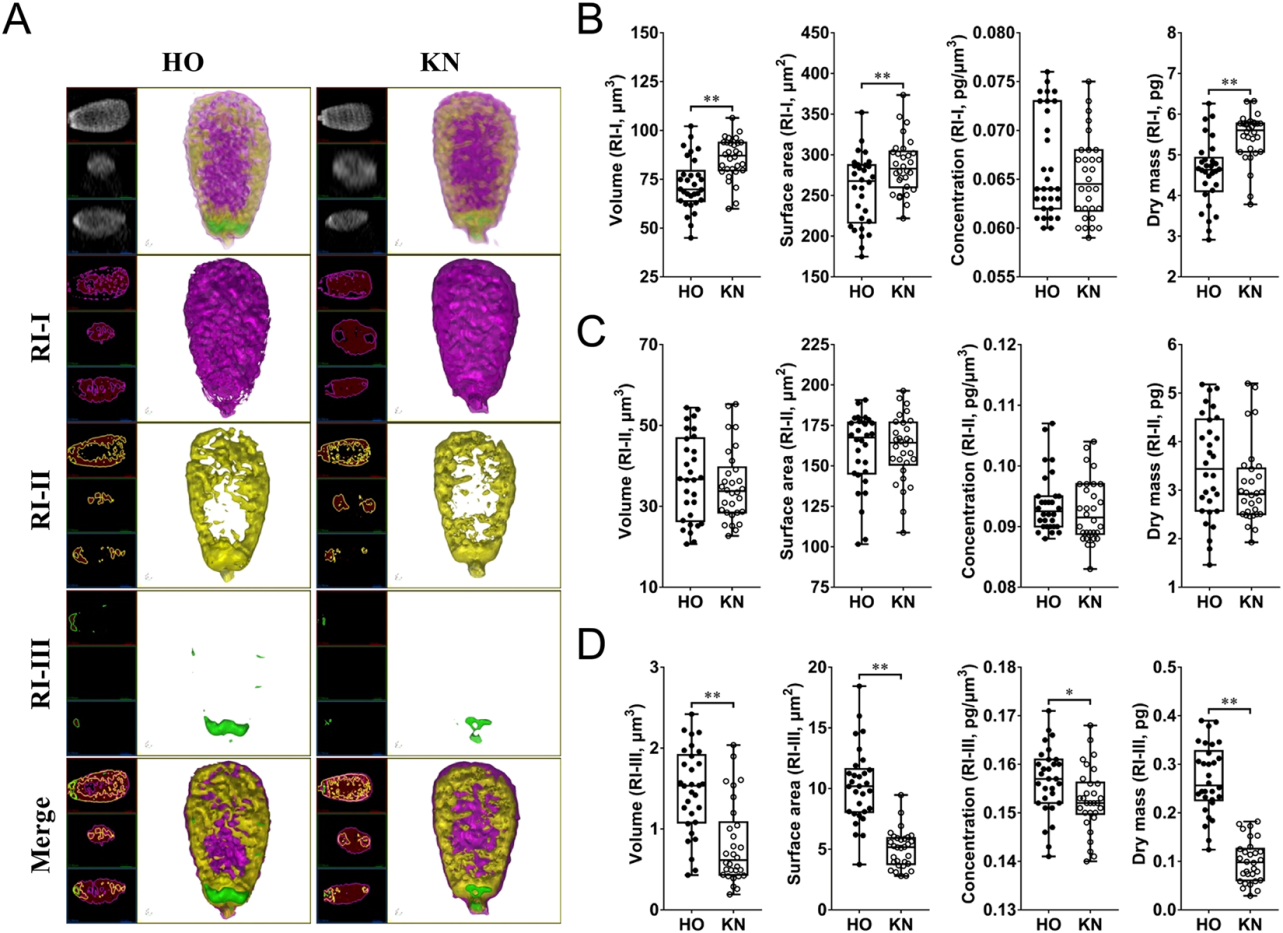
Substances distribution and measurements of HO and KN sperm head based on different RI. (**A**) 3D RI tomograms of HO and KN sperm heads at different RI ranges. Substances belonging to RI-I, RI-II, and RI-III are labelled in purple, yellow, and green, respectively. (**B**,**C**,**D**) The volume, surface area, concentration, and dry matter mass of the sperm head.

**Figure 4:**
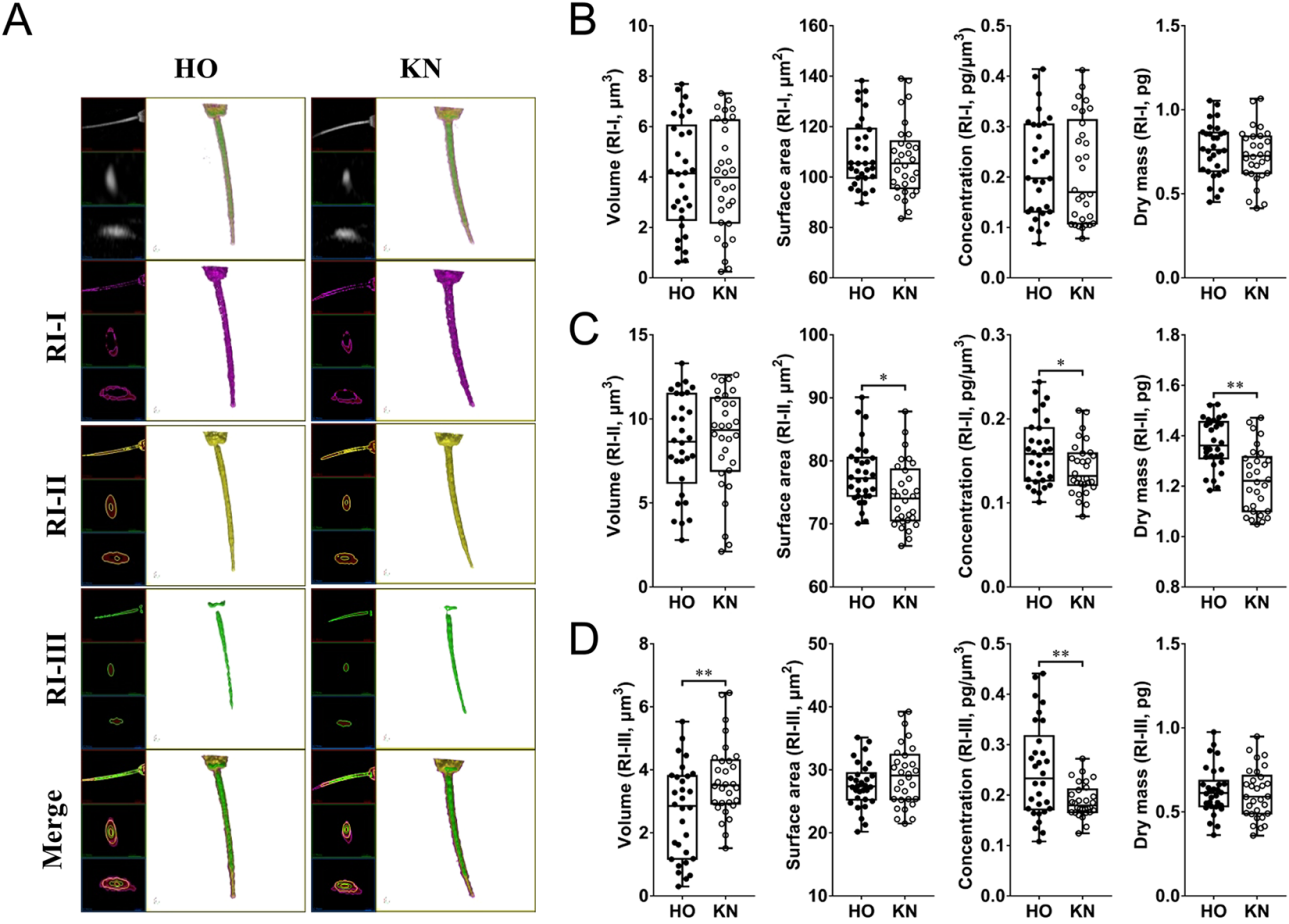
Substances distribution and measurements of HO and KN sperm midpiece based on different RI. (**A**) 3D RI tomograms of HO and KN sperm midpiece at different RI ranges. Substances belonging to RI-I, RI-II, and RI-III are labelled in purple, yellow, and green, respectively. (**B–D**) The volume, surface area, concentration, and dry matter mass of the sperm midpiece.

**Figure 5:**
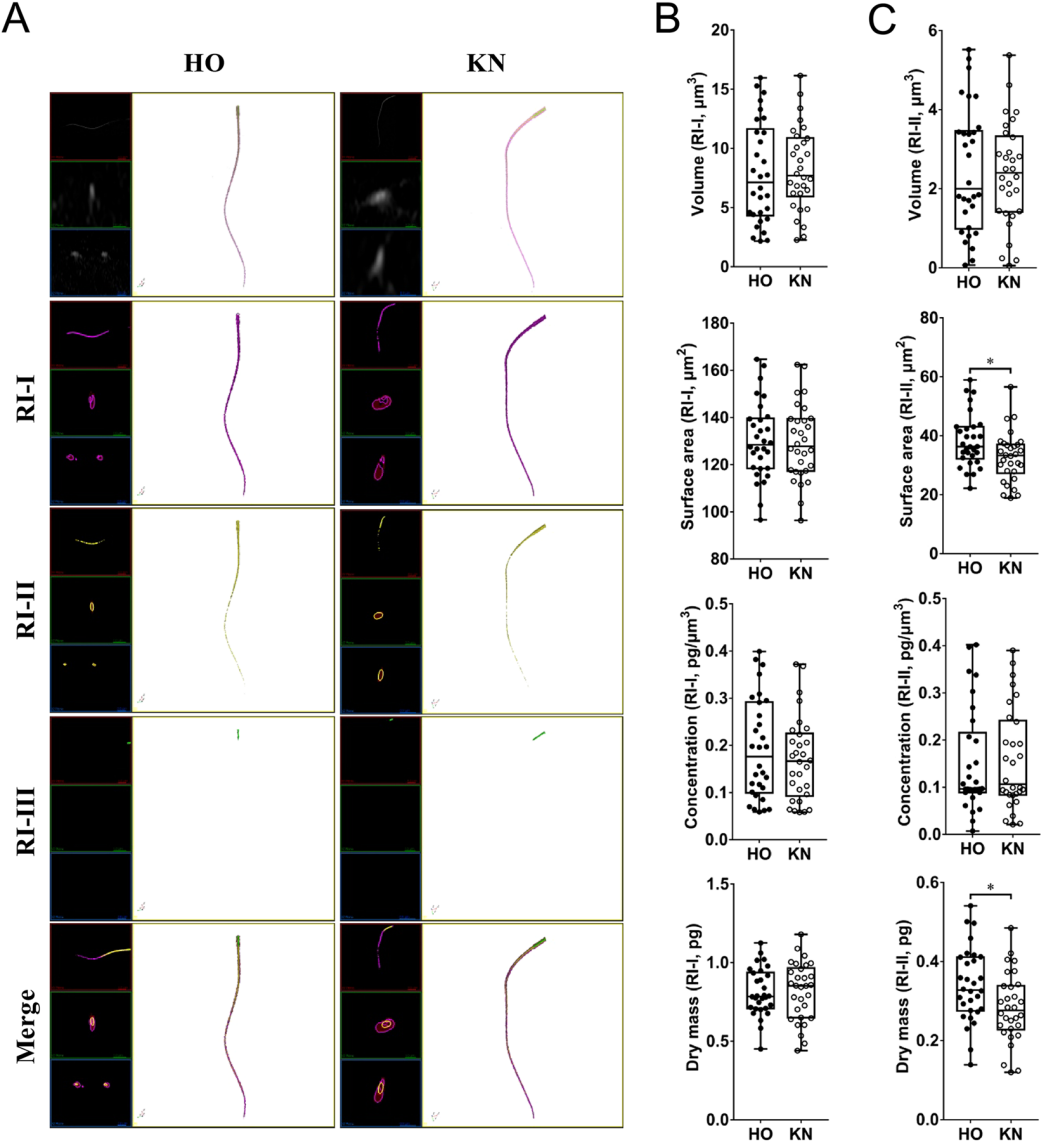
Substances distribution and measurements of HO and KN sperm tail based on different RI. (**A**) 3D RI tomograms of HO and KN sperm tails at different RI ranges. Substances belonging to RI-I, RI-II, and RI-III are labelled in purple, yellow, and green, respectively. (**B–D**) The volume, surface area, concentration, and dry matter mass of the sperm tail.

After we confirmed that we could accurately and specifically distinguish substances with different RI parameters, we utilized the same analytical parameters (i.e., RI-I, RI-II, and RI-III) to show that the head (Fig. 3 and Video 7), midpiece (Fig. 4 and Video 8), and tail (Fig. 5 and Video 9) of the HO and KN spermatozoa exhibited significant differences. As shown in Table 2, RI-I range substances were significantly different in volume, surface area, and dry mass of the sperm head between HO cows and KN cattle. RI-II range substances were significantly different in surface area, concentration, and dry mass of the midpiece between HO cows and KN cattle, as well as the surface area and dry mass of the tail. RI-III range substances were significantly different in volume, surface area, concentration, and dry mass of the sperm head between HO cows and KN cattle, as well as the volume and concentration of the midpiece.

**Table 2:** The 3D Morphology differences of sperm between HO and KN with different RI ranges.

Subpart	RI range	Species	Volume (μm^3^)	Surface area (μm^2^)	Concentration (fg/μm^3^)	Dry mass (pg)
Head	RI-I	HO	72.42 ± 2.45^A^	257.40 ± 7.88^A^	66.53 ± 0.98	4.57 ± 0.15^A^
		KN	85.66 ± 1.93^B^	286.40 ± 6.17^B^	65.13 ± 0.78	5.44 ± 0.11^B^
	RI-II	HO	36.95 ± 1.928	159.60 ± 4.25	93.77 ± 0.89	3.48 ± 0.19
		KN	35.11 ± 1.62	161.10 ± 3.71	92.57 ± 0.95	3.09 ± 0.15
	RI-III	HO	1.48 ± 0.10^A^	10.25 ± 0.57^A^	156.60 ± 1.27^a^	0.27 ± 0.01^A^
		KN	0.81 ± 0.09^B^	5.02 ± 0.28^B^	152.70 ± 1.21^b^	0.10 ± 0.01^B^
Midpiece	RI-I	HO	4.06 ± 0.40	109.40 ± 2.47	218.0 ± 17.83	0.76 ± 0.03
		KN	3.97 ± 0.39	106.90 ± 2.66	210.2 ± 19.00	0.73 ± 0.03
	RI-II	HO	8.49 ± 0.54	77.91 ± 0.89^a^	160.1 ± 7.13^a^	1.37 ± 0.02^A^
		KN	8.80 ± 0.54	74.54 ± 0.95^b^	140.8 ± 5.69^b^	1.23 ± 0.02^B^
	RI-III	HO	2.66 ± 0.27^A^	27.60 ± 0.66	248.5 ± 17.24^A^	0.63 ± 0.03
		KN	3.68 ± 0.22^B^	29.10 ± 0.89	188.5 ± 6.04^B^	0.61 ± 0.01
Tail	RI-I	HO	7.92 ± 0.79	130.30 ± 2.98	191.9 ± 19.53	0.81 ± 0.03
		KN	8.26 ± 0.64	129.50 ± 2.93	170.4 ± 16.23	0.82 ± 0.03
	RI-II	HO	2.48 ± 0.29	38.27 ± 1.64^A^	151.7 ± 20.16	0.34 ± 0.02^A^
		KN	2.38 ± 0.24	32.72 ± 1.55^B^	156.9 ± 19.59	0.28 ± 0.02^B^
	RI-III	HO	—	—	—	—
		KN	—	—	—	—

Significant differences are represented with different lower-case letters (P < 0.05) and different capital letters (P < 0.01) between HO and KN. — Represents undetectable.

**Figure 6:**
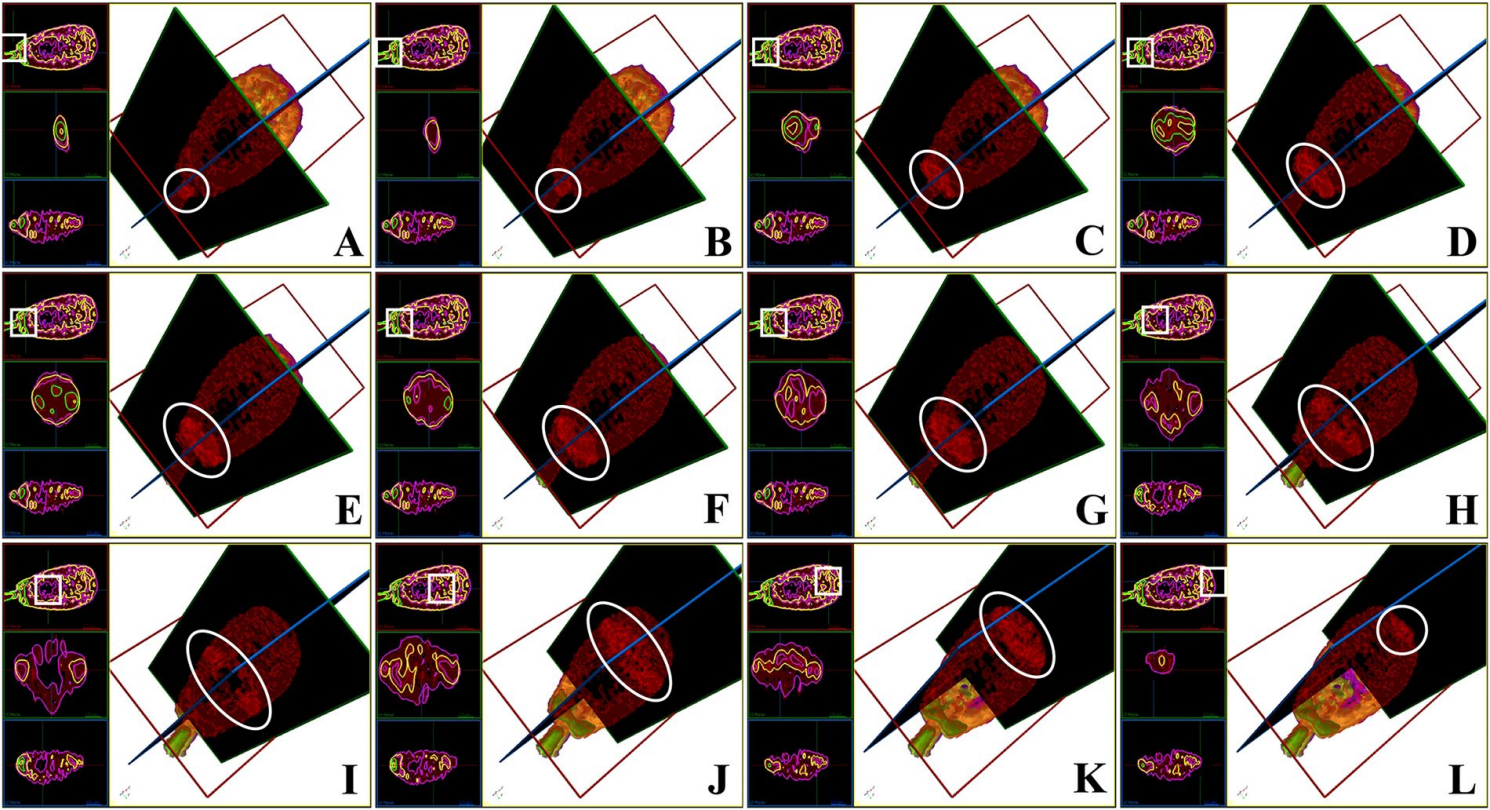
3D structure of the sperm head based on RI-I, RI-II, and RI-III. (**A**) to (**L**) Cross-sectional images of the RI tomogram of a representative sperm at various positions inside the head. The squares and circles represent the observed location. Substances belonging to RI-I, RI-II, and RI-III are labelled in purple, yellow, and green, respectively. The images with black background represent the perspective views and indicate the corresponding cross-sections shown in each figure.

**Figure 7:**
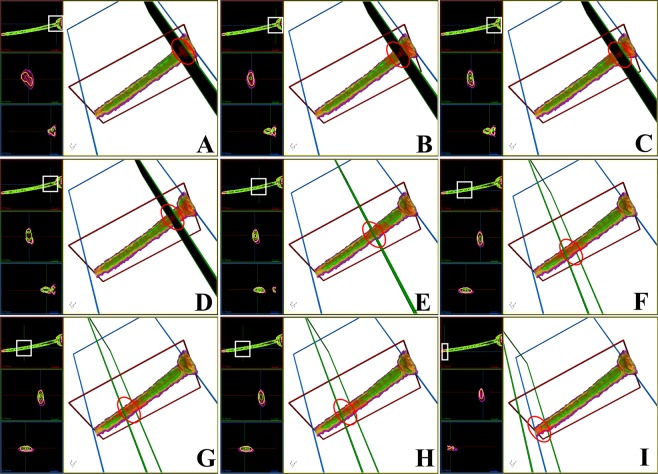
3D RI tomograms of a sperm midpiece based on RI-I, RI-II, and RI-III. (**A**) to (**I**) Cross-sectional images of the RI tomogram of a representative sperm at various positions inside the midpiece. White squares and red circles represent the observed location. Substances belonging to RI-I, RI-II, and RI-III are labelled in purple, yellow, and green, respectively. The images with black background represent the perspective views and indicate the corresponding cross-sections shown in each figure.

**Figure 8:**
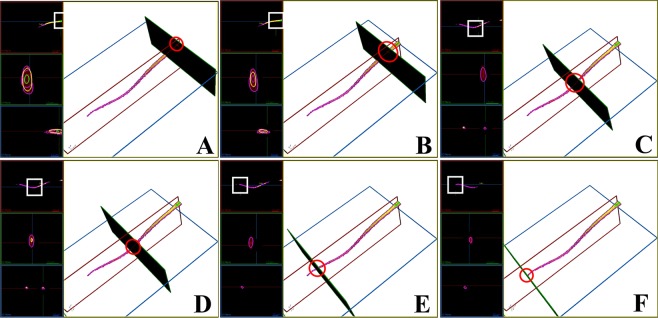
3D structure of a sperm tail on RI-I, RI-II, and RI-III. (**A**) to (**I**) Cross-sectional images of the RI tomogram of a representative sperm at various positions inside the tail. White squares and red circles represent the observed location. Substances belonging to RI-I, RI-II, RI-III are labelled with purple, yellow, and green, respectively. The images with black background represent the perspective views and indicate the corresponding cross-sections shown in each figure.

In general, the substances with different RIs of the entire spermatozoa exhibited a bilateral symmetry distribution, not a radial symmetry distribution (Figs 2, 6–8). The composition of the entire spermatozoa substance was generally the same as that of the tail, except for the midpiece that contained regions with high RI. The midpiece and the portion linked to the head contained a substance within the RI-II range.

In the sperm head (Fig. 6 and Video 10), substances in the RI-I range were mainly distributed on the surface of the sperm, and substances in the RI-II range constituted the “skeletal system” of the sperm head (Fig. 3, Supplementary Fig. S3A and Supplementary Fig. S3B). Most of the substances in the RI-III range were only located at the bottom of the sperm head (near the midpiece). However, the substances in the RI-III range were also detected on both sides of the head, and in the acrosome position in a few spermatozoa (Supplementary Fig. S3C and Supplementary Fig. S3D). Specifically, the RI-II substances started from the bottom of the head and gradually decreased toward the acrosome until they were completely undetectable (Fig. 6A–G). Throughout this disappearing process, RI-II substances first decreased on the back and abdomen of the sperm head (Fig. 6D,E), and then disappeared from both sides (Fig. 6F,G).

RI-II range substances were distributed continuously; however, they did not exhibit an absolute ring-shaped structure. When their position reached approximately 1/5 of the length from the bottom of the sperm head, a cavity-like region with a relatively low RI value was observed, which extended toward the top of the head (Fig. 6H–J). This cavity reached approximately 2/5 of the length from the top of the sperm head. The spermatozoon acrosome was mainly composed of substances in the RI-II range (Fig. 6K,L). The size of the cavity varied among different sperm cells (Video 10, Supplementary Fig. S3E–G).

The substance in the RI-III range was widely distributed in the midpiece. Although some of the substances were distributed in the head, no substance in the RI-III range was detected at the linkage position of the midpiece and the head (Fig. 7A). The substance in the RI-II range was separated from the substance in the RI-III range (Fig. 7C,E and G) and exhibited a discontinuous distribution (Fig. 7B–H). The substance in the RI-III range disappeared at the end of the midpiece (Fig. 7I and Video 11).

From the end of the spermatozoan midpiece to the tail, there was no positional distribution of RI-III (Fig. 8B–F), and the components that constitute the sperm tail belonged mainly to RI-I and RI-II. We also found a case where the RI-II range substance was intermittently distributed (Fig. 8B–D) and could no longer be detected at the end of the tail (Fig. 8E,F; Video 12).

## Discussion

The morphology and internal structure of spermatozoa have a great influence on their motility, affecting slither swimming mode, oocyte fertilization rate, and non-return rates, and are affected by environmental factors, genetics, and the physiological state of individuals^30^. The evaluation of spermatozoa quality has been highlighted as a predictor of fertilization success^31^. Although they can provide valuable information regarding the overall cell shape, 2D label-free imaging methods can only detect abnormal spermatozoa with significant morphological changes and incomplete structures; other methods are mostly based on the chemical labeling of specific substances.

Recently, quantitative phase and label-free imaging techniques have been successfully applied to spermatozoan imaging and digital structure construction. Similar to other studies, the present approach provided a label-free 3D imaging method, which could noninvasively measure the biochemistry and morphology of spermatozoa cells. The physiological state of the spermatozoa was not altered, yet both the external and internal structures could be comprehensively analyzed. QPI techniques are suitable for quick detection and imaging while correlative spectroscopy imaging (like Raman spectroscopy) is good at molecular characterization^32^. For example, Raman spectroscopy can reliably and independently detect the activation state of cells, and provides information at the molecular level, allowing for the investigation of functional groups, bonding types, and molecular conformations, and correlating them to the cell structural properties^33,34^. Utilizing QPI techniques, the present study not only acquired the full 3D structure of the studied bovine spermatozoa, but also revealed their 3D translational head motion and the angular velocity of their head spin as well as the 3D flagellar motion and the preference of bovine spermatozoa for helix-shaped 3D swimming trajectories^35,36^. Moreover, the substance distribution in spermatozoa was distinguished accurately and specifically with a 0.005 gradient range. Therefore, we were able to use specific RIs to identify the distribution of related substances and to determine substances in an abnormal state.

Studies have shown that the length of the sperm midpiece is significantly different among different breeds of cattle with different feeding purposes such as beef cattle or dairy cows, and there are large differences among individuals within each breed^37^. Previous studies have shown that a sperm midpiece possesses a plasma membrane, mitochondrion, dense fibers, doublet, central sheath, tubules, and other important organelles from the outside to the inside^38^. In the present study, we found that the RI-III range substance was widely and almost uniquely distributed in the midpiece. The RI-II range substance was separated by the RI-III range substance and exhibited discontinuous distribution; therefore, we hypothesized that the RI-III range substance was a mitochondria-related substance, which was mainly distributed in the midpiece with tyrosine phosphorylated proteins, two of which are phospholipid hydroperoxide glutamate peroxidize and ATP synthase beta subunit, and play a primary role in spermatozoan motility^39^. Undoubtedly, structural abnormality of the sperm midpiece has a negative effect on the spermatozoan quality. In the present study, a combination of 2D and QPI imaging was used to analyze the spermatozoa midpiece. We first identified the different midpiece lengths of the HO cow and KN cattle spermatozoa. Then, the different volumes and distributions of the spermatozoa in the two breeds were measured with different RIs. The results showed that the corresponding mitochondrial distribution range (or volume) might also be different. Therefore, there are potential differences between HO cow and KN cattle spermatozoa motility, patterns, and adaptation to the environment. Our analysis provides the basis for vibrant sperm cell selection in artificial insemination and *in vitro* fertilization. However, whether and how the length and inner structure of the midpiece is meaningful for animal breeding remains unclear.

An abnormal spermatozoon head is often caused by genetic defects^40^, food intake^41^, chemical substances, or radio-frequency radiation^42^, which results in a loss of fertilization ability. Simultaneously, conventional staining and labeling-based imaging techniques may change the morphometric dimensions of a spermatozoon head, with the plasma membrane, acrosome, and nucleus being the most important components^43^. Therefore, a QPI technique that can shorten the analysis time and maintain the morphology and structural integrity of a spermatozoon head would be highly beneficial for subsequent artificial insemination and assisted reproductive technology. The present study found that the RI distribution inside the spermatozoon head is complicated, and there were significant differences in the spermatozoa between the two bovine breeds, especially in the nucleus and acrosome. First, the substance corresponding to the same RI exhibited a discontinuous distribution along the x-y, y-z, and x-z planes inside the head. These findings are similar to previous studies that showed the existence of spermatozoa chromatin positioning factors specific to individual chromosomes. The nucleus can be divided into several asymmetric areas. These non-random preferential lateral and longitudinal intranuclear positionings have potential effects on the fertilization process and chromatin remodeling events during the early stages of embryogenesis^44^. Second, there are differences in the nuclear space of different bovine breeds and the same RI distribution inside them. Whether this affects the ability of spermatozoa to achieve insemination between breeds is unclear. However, this might be a new direction and new ideas for screening the morphology, function, and quality of the spermatozoon head.

The present method can potentially be used in various applications, including sperm quality monitoring and rapid selection. It can also provide information regarding unexplained failures of multiple assisted reproductive techniques and recurrent abortions. For example, the general morphology and structure of spermatozoa are known to be related to sperm motility and competition ability^45,46^. However, one limitation of the present method is a lack of molecular specificity. Unlike fluorescent labeling techniques, RI distribution cannot provide specific molecular information because the RI increments that are necessary to convert RI values to the local concentration of intracellular components are too similar^28^. Although the molecular specificity of the present RI measurement technique may not be as high as labeling methods, it could be improved by measuring spectroscopic RI distributions, which can separate molecules spectrally owing to their different optical dispersion properties^47^. We also performed measurements of the dry mass at the cellular and subcellular level. This approach can be utilized for animal reproduction and human assisted reproduction, especially spermatozoa selection during preparation for artificial insemination^8^.

In summary, we presented the measurements of 3D RI tomograms of individual spermatozoon and performed a systematic analysis of the morphology and internal structures of spermatozoa from HO cows and KN cattle. The results showed that HO cow and KN cattle spermatozoa have significant differences in morphological parameters such as length, width, and volume of the head, midpiece, and tail. There were also significant differences in the distribution of intracellular components containing the same RI inside the head, midpiece, and tail. These results provide new techniques for further in-depth structural analysis, quality monitoring, and spermatozoa selection in a rapid and label-free manner. Whether the internal RI distribution of spermatozoa can be used as a diagnostic parameter to evaluate the state of a spermatozoa and fertilization ability remains an open question; however, it is now accessible to direct experimental study.

## Materials and Methods

### Ethics statement

All methods (including all animal experiments) in this study were performed in accordance with the relevant guidelines and approved by the Institutional Committee on the Use and Care of Chungbuk National University (CBNUR-1026–16).

### Principles of optical diffraction tomography

To measure 3D RI tomograms of individual spermatozoon, we used a commercial ODT system (HT-1H, Tomocube Inc., Republic of Korea)^48^. CT reconstructs X-ray absorptivity in 3D, whereas ODT reconstructs RI distributions using information regarding light absorptivity and RI. ODT reconstructs the 3D RI tomogram of a sample from the measurements of multiple 2D holograms with various illumination angles^28^. The experimental setup is based on an off-axis Mach-Zehnder interferometer equipped with a digital micromirror device (DMD)^49,50^ (Supplementary Fig. S4).

### Sample collection and preparation

Mouse spermatozoa were isolated from two-month-old ICR mice. Longitudinal cuts were made in the cauda epididymis and the tissue was incubated in phosphate-buffered saline (PBS) solution at 37 °C to enable motile, mature spermatozoa to swim out. The Large White pig semen samples were obtained from boars in the local farm (Darby genetics, Anseong, Gyeonggi-do, Korea) using the gloved-hand method and delivered to the laboratory immediately under 37 °C. Semen samples were washed three times with 0.85% NaCl (w:v) containing 100 mg/L bovine serum albumin. Frozen semen from five Holstein (HO, a well-known dairy cattle) bulls and five Korean native (KN, a beef cattle with good meat quality) bulls were obtained from local farms (Nonghyup Economic Landholding Hanwoo Improvement offices, Chungnam, Korea). Their spermatozoa were separated using the Percoll method^51^, gently washed with PBS, and the number of sperm was adjusted to a density suitable for observation. One drop (approximately 25 µL) of PBS containing spermatozoa from the different breeds was placed on a 0.13–0.17 mm NEO micro glass slide (Matsunami, Tokyo, Japan) and measured using the HT-1H system. Six spermatozoa from each semen sample were randomly selected for observation.

### Image acquisition and 3D RI model reconstruction

For tomographic reconstruction, a total of 30 holograms of the single spermatozoon sample at various illumination angles were measured. Using a phase retrieval algorithm^52,53^, optical field images containing both the amplitude and phase maps of the sample were retrieved from the measured holograms. Based on the Fourier diffraction theorem with Rytov approximation^54^, the 3D RI tomogram of the sample was reconstructed from the multiple 2D optical field images. The lateral and axial optical resolutions of the ODT system were 110 nm and 360 nm, respectively, according to the Lauer criterion^55,56^. Due to the limited numerical apertures of the condenser and objective lenses, side scattering information was not retrieved, resulting in inaccuracy in tomographic reconstruction, the so-called “missing cone” problem. To remedy this, an iterative regularization algorithm based on non-negativity was applied to the reconstruction process^57^.

### Quantitative image analysis

The structures of the head, midpiece, and tail of HO and KN spermatozoon cells were systematically investigated by retrieving morphological properties from the measured 3D RI maps. In each 3D RI map, the cell and subcellular compartments were determined from the RI values. The RI range (RI_start-end_) in spermatozoa cells was divided into three groups (RI-I: 1.3451-1.3520; RI-II: 1.3521-1.3640; and RI-III: 1.3641−1.382). For visualization, substances belonging to RI-I, RI-II, or RI-III were digitally color-coded with purple, yellow, or green, respectively, unless stated otherwise. The volumes of the whole spermatozoa cells and their compartments (head, midpiece, and tail) were retrieved by multiplying the unit volume of a voxel by the total number of voxels in the occupied region. The surface area was calculated as the area of the outermost surface of the occupied region. The sphericity index of each spermatozoon head was calculated from the volume and surface area. A higher sphericity index indicated a stronger resemblance to a sphere.

The concentration of dry matter mass in a spermatozoon sample can be calculated from its RI value because the cytoplasm protein and lipid concentrations are linearly proportional to their RI value^29,58^ by a constant called the refractive index increment. Most non-aqueous small molecules in the spermatozoa, including proteins, DNA, and lipids, have similar RII values^59^. The dry mass of the occupied region in sperm cells was then calculated by multiplying the dry mass concentration by the occupied volume.

### Statistical analysis

Results are presented as the mean ± standard deviation (SD). Data obtained from two groups were compared using Student’s *t*-test. All statistical analyses were performed using SPSS version 22.0 (IBM, IL, USA) software. Solid dots and open circles represent HO and KN measurements, respectively. Significant differences are represented with *(P < 0.05) and **(P < 0.01).

## Supplementary information

Supplementary Information

Normal movements of bovine sperm.

Distorted and rotary movement of abnormal sperm.

Twirling round movement of abnormal sperm.

Movements of abnormal sperm with low vitality

Abnormal movement of two sperm with head sticking together.

Abnormal movement of sperm.

Reconstruction of bovine sperm head.

Reconstruction of bovine sperm midpiece.

Reconstruction of bovine sperm tail.

Structure scanning of sperm head.

Structure scanning of sperm midpiece.

Structure scanning of sperm tail.
