# Supplementary material for: Reconstruction of bovine spermatozoa substances distribution and morphological differences between Holstein and Korean native cattle using three-dimensional refractive index tomography
Source: Sci Rep. 2019 Jun 19;9:8774. doi: 10.1038/s41598-019-45174-3 (PMC6584538; doi:10.1038/s41598-019-45174-3)
Supplement: Supplementary file 1 — Supplementary Information [file 41598_2019_45174_MOESM1_ESM.docx]

**Supplementary Information**

**Reconstruction of bovine spermatozoa substances distribution and morphological differences between Holstein and Korean native cattle using three-dimensional refractive index tomography**

Hao Jiang^1,5^, Jeong-woo Kwon^1^, Sumin Lee^2^, Yu-Jin Jo^1^, Suk Namgoong^1^, Xuerui Yao^1^, Bao Yuan^5^, Jia-bao Zhang^5^, Yong-Keun Park^2,3,4^, Nam-Hyung Kim^1,5,*^

1 Department of Animal Sciences, Chungbuk National University, Cheong-Ju, Chungbuk, Republic of Korea.

2 Tomocube, Inc., 48, Yuseong-daero 1184beon-gil, Yuseong-Gu, Daejeon 34051, Republic of Korea

3 Department of Physics, Korea Advanced Institute of Science and Technology (KAIST), Daejeon 34141, Republic of Korea

4 KAIST Institute for Health Science and Technology, KAIST, Daejeon 34141, Republic of Korea

5 College of Animal Science, Jilin University, Changchun, China

**Correspondence:**

Nam-Hyung Kim, Ph.D.

Department of Animal Sciences, Chungbuk National University, Cheong-Ju, Chungbuk, Republic of Korea.

Phone: +82-42-3261-2546

Email: nhkim@chungbuk.ac.kr


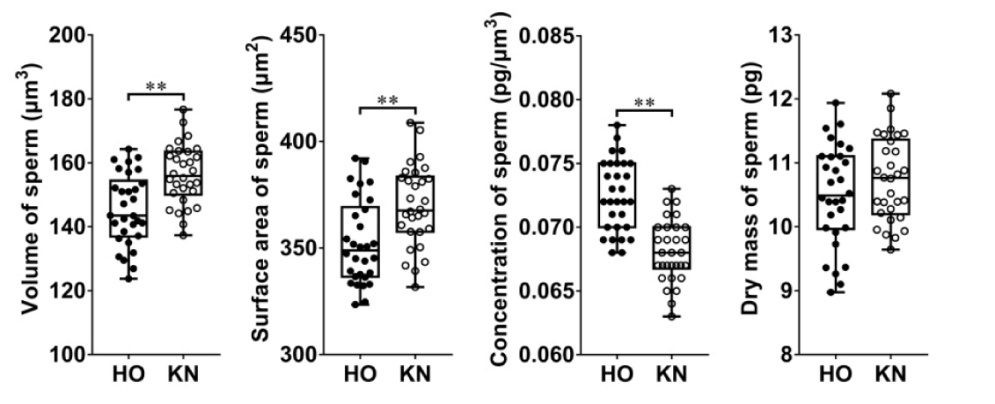


**Supplementary Figure S1. Morphology differences of whole sperm between HO and KN.**


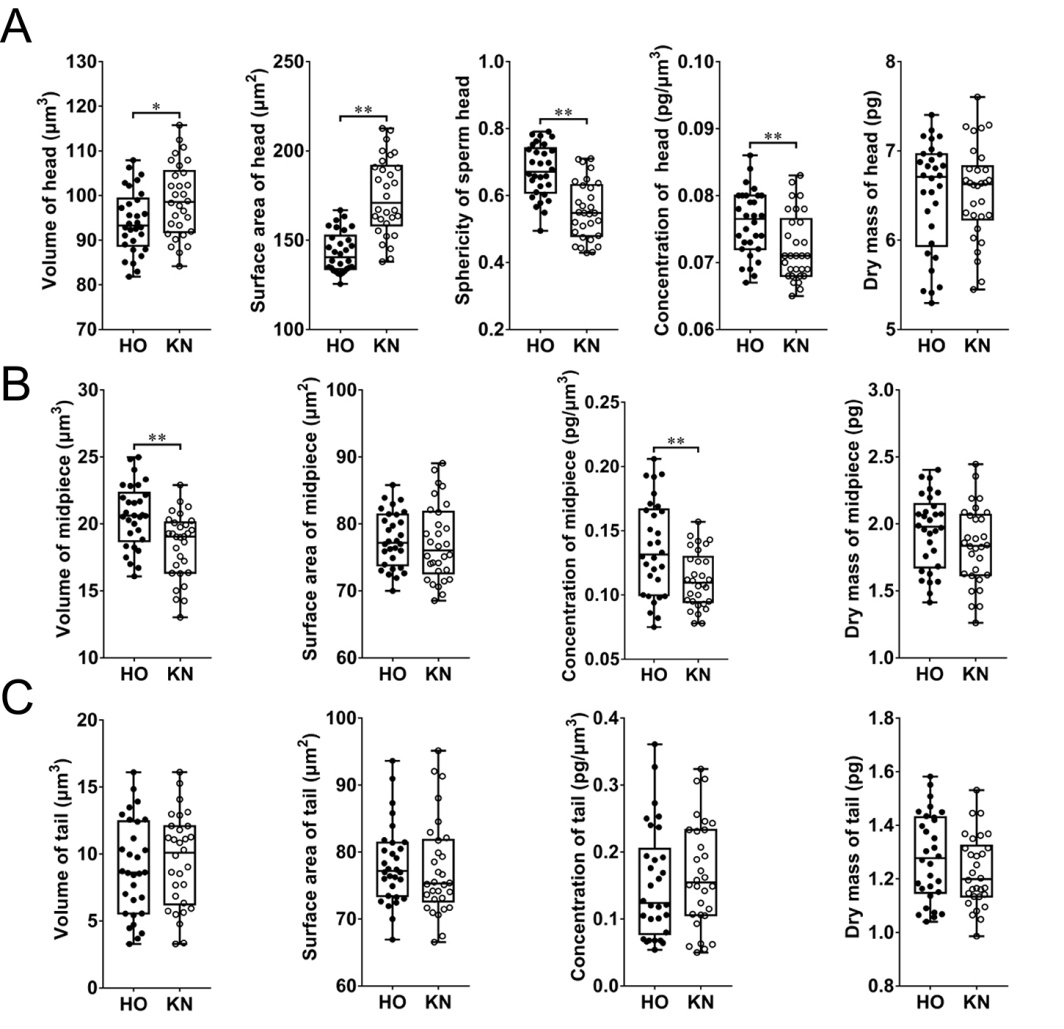


**Supplementary Figure S2. General 3D morphology differences of head, midpiece,and tail between HO and KN.** Volume, surface area, sphericity, concentration and dry matter mass of sperm head (A), sperm midpiece (B), and sperm tail (C).


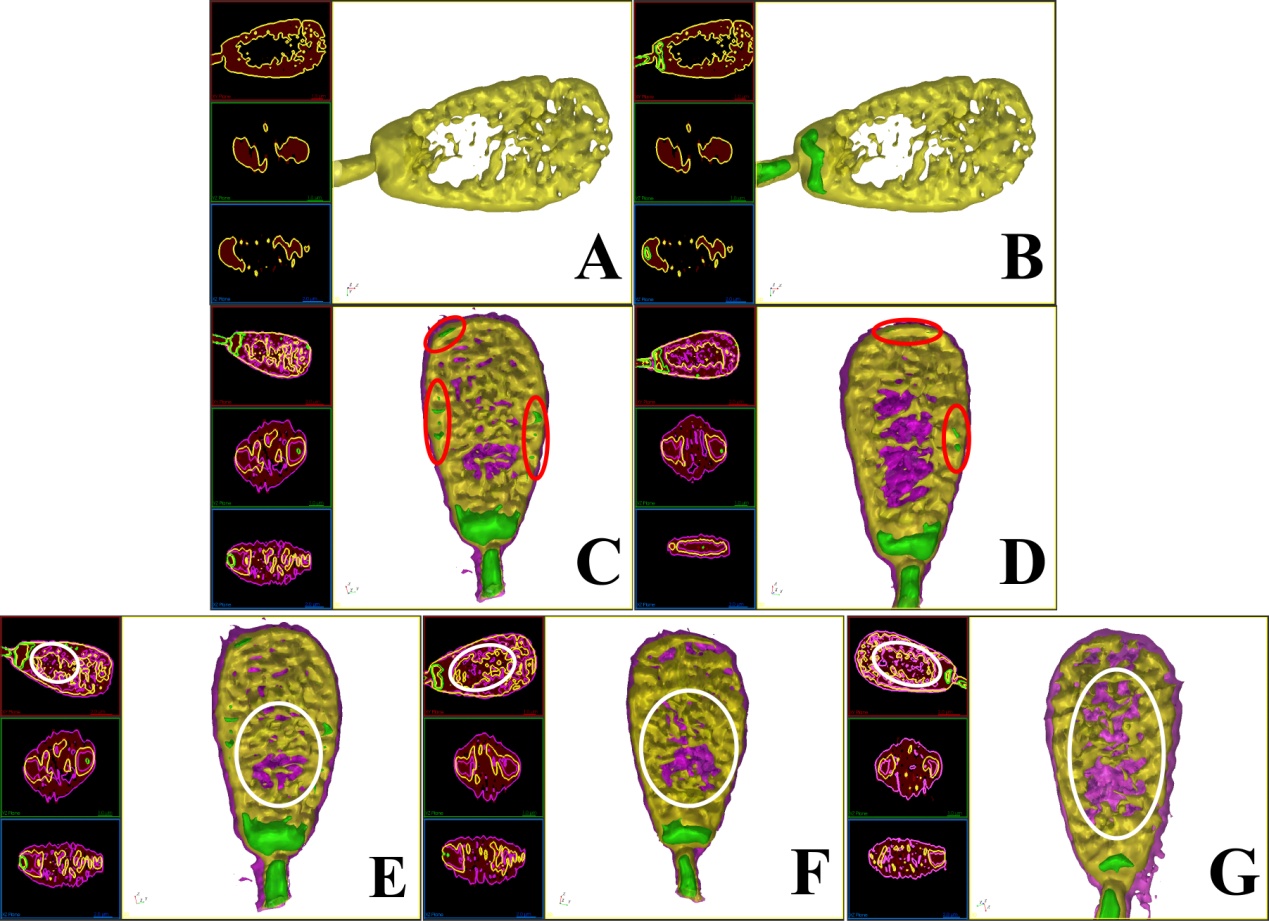


**Supplementary Figure S3. RI tomograms of a sperm head.** White and red circles represent the observed location. Substances belonging to RI-I, RI-II, and RI-III are labeled with purple, yellow, and green, respectively. The pictures with a black background on the left side of each image represent the cross-sectional images of the RI tomogram along the x-y, y-z, and x-z axis of the observed field. A: Substances belonging to RI-I* (1.3521 to 1.382) are labeled in yellow. B: Substances belonging to RI-II* (1.3521-1.364) and RI-III (1.3641-1.382) are labeled in yellow and green, respectively. There is hardly any difference between substances labeled with RI-II and RI-I (1.3521 to 1.382). C and D: The sperm with the substance belonging to RI-III distributed on the side and top (red circles) of the sperm head. E to F:The different sizes of the cavity. White circles represent the observed cavity.


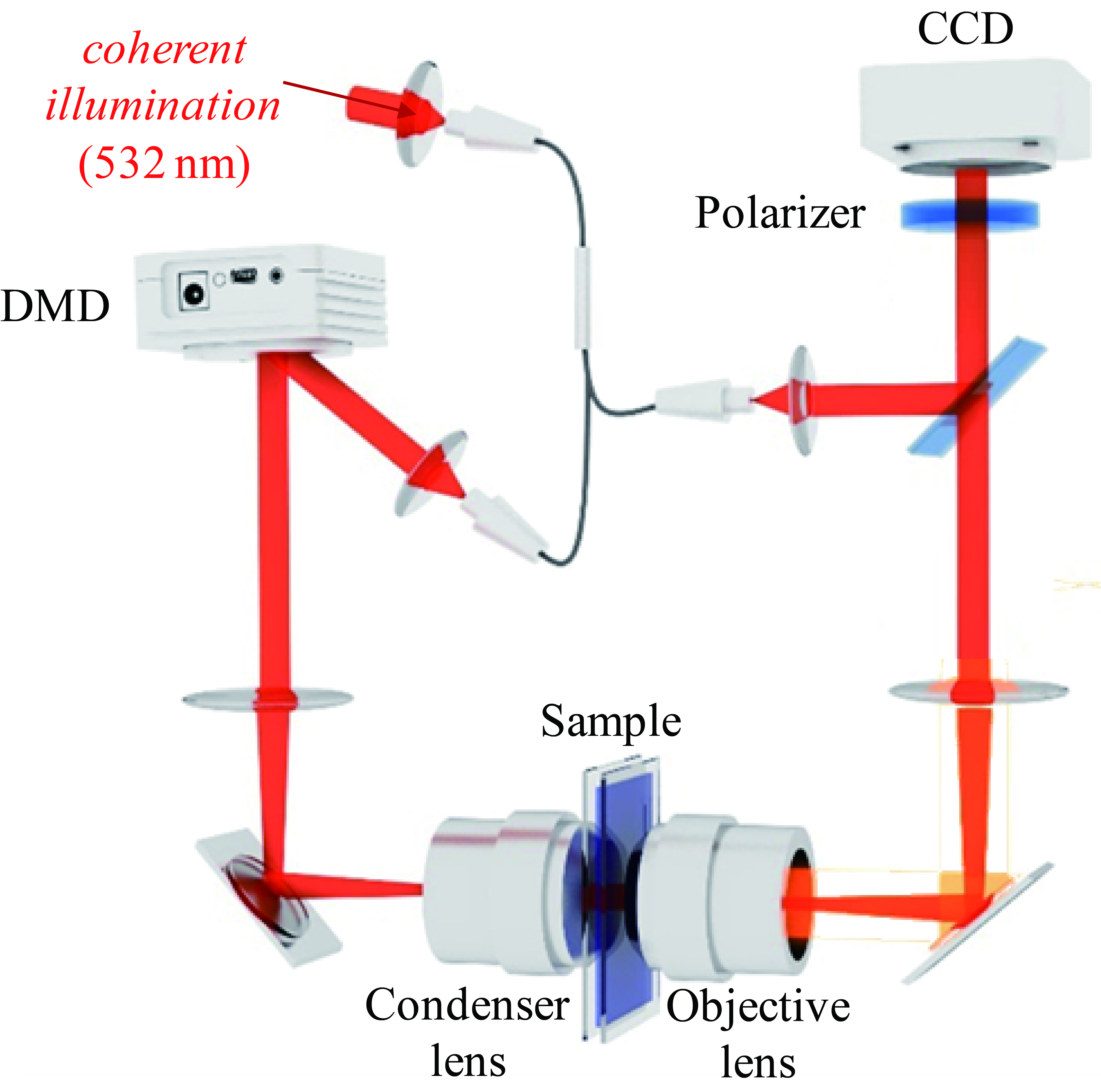


**Supplementary Figure S4. Optical setup.** A laser beam from a coherent, monochromatic laser (wavelength, *λ* = 532 nm) was split into a sample and a reference arm using a 2×2 optical fiber coupler. To control the illumination angle of the beam impinging onto a sample, a sample beam is diffracted from a DMD in which holograms patterns were displayed. Then, the beam scattered by the sample was collected using an objective lens (60×, numerical aperture = 1.2, water immersion), and projected onto an image sensor. At the sensor plane, the sample and the reference beam interfere with a slight tilt angle, resulting in the formation of a spatially modulated hologram.

**Supplementary Table S1.**  **Measurement of single sperm volume, surface area, concentration and dry based on the different RI range.**

| RI range | Volume (um^3^)  (CV%) | Surface area (um^2^)  (CV%) | Concentration (pg/um^3^)  (CV%) | Dry mass (pg)  (CV%) |
| --- | --- | --- | --- | --- |
| 1.345-1.350 | 77.0134  (0.00067) | 464.0135  (0.00011) | 0.0594  (0.86936) | 4.6438  (0.00908) |
| 1.350-1.355 | 73.0905  (0.00073) | 291.3904  (0.00017) | 0.0786  (0.65700) | 5.8034  (0.00890) |
| 1.355-1.360 | 20.4126  (0.00253) | 162.8663  (0.00030) | 0.1043  (0.46313) | 2.1445  (0.02457) |
| 1.360-1.365 | 6.4965  (0.00812) | 58.5855  (0.00090) | 0.1374  (0.37584) | 0.8947  (0.05399) |
| 1.365-1.370 | 2.5207  (0.01916) | 27.8008  (0.00152) | 0.1634  (0.31603) | 0.4115  (0.12808) |
| 1.370-1.375 | 0.5524  (0.09348) | 8.0735  (0.00653) | 0.1905  (0.27667) | 0.1046  (0.49369) |
| 1.375-1.380 | 0.1206  (0.42819) | 2.3225  (0.02269) | 0.2226  (0.23199) | 0.0263  (1.83668) |

Note: CV refers to Coefficient of Variation.
